# Supplementary material for: New Insights into the Estimation of Reproduction Numbers during an Epidemic
Source: Vaccines (Basel). 2022 Oct 25;10(11):1788. doi: 10.3390/vaccines10111788 (PMC9694736; doi:10.3390/vaccines10111788)
Supplement: Supplementary file 1 [file vaccines-10-01788-s001.zip › vaccines-1944616-supplementary.pdf]

Article

Supplementary Material for "New Insights to Estimate Reproduction Numbers during an Epidemic"

We derive here Equation (12) of the main text. For any time $t = 1, \ldots, T$, we get

$$\begin{aligned} Y(t) &= R_t(t)(Y * f_C)(t) \\ &= R_t(t) \sum_{k=1}^{t} f_C(t-k)Y(k) + R_t(t) \sum_{k=-\infty}^{0} f_C(t-k)Y(k) \\ &= R_t(t) \sum_{k=1}^{t} f_C(t-k)Y(k) + R_t(t) \sum_{k=-\infty}^{0} f_C(t-k) \\ &= R_t(t) \sum_{k=1}^{t} f_C(t-k)Y(k) + R_t(t) \left[1 - \sum_{x=0}^{t-1} f_C(x)\right]. \end{aligned}$$

By solving with respect to $Y(t)$, we obtain

$$Y(t) = \frac{R_t(t) \sum_{k=1}^{t-1} f_C(t-k)Y(k)}{1 - R_t(t) f_C(0)} + \frac{R_t(t) \left[1 - \sum_{x=0}^{t-1} f_C(x)\right]}{1 - R_t(t) f_C(0)}. \quad (12)$$

We now provide the explicit computations to derive the standard estimator $\hat{R}_t(0)$ given in (13) of the main text.

$$\begin{aligned} \hat{R}_t(0) &= \frac{\sum_{i=-7}^{6} R_t(i)(Y * f_C)(i)}{\sum_{i=-7}^{6} (Y * f_C)(i)} \\ &= \frac{R_t(0) \sum_{i=-7}^{0} (Y * f_C)(i) + \sum_{i=1}^{6} R_t(i)(Y * f_C)(i) \pm R_t(0) \sum_{i=1}^{6} (Y * f_C)(i)}{\sum_{i=-7}^{6} (Y * f_C)(i)} \\ &= \frac{R_t(0) \sum_{i=-7}^{6} (Y * f_C)(i) + \sum_{i=1}^{6} R_t(i)(Y * f_C)(i) - R_t(0) \sum_{i=1}^{6} (Y * f_C)(i)}{\sum_{i=-7}^{6} (Y * f_C)(i)} \\ &= R_t(0) + \frac{\sum_{i=1}^{6} [R_t(i) - R_t(0)](Y * f_C)(i)}{\sum_{i=-7}^{6} (Y * f_C)(i)} = R_t(0) + \frac{\sum_{i=1}^{6} [R_t(i) - R_t(0)] \frac{Y(i)}{R_t(i)}}{\sum_{i=-7}^{6} \frac{Y(i)}{R_t(i)}}. \quad (13) \end{aligned}$$
